# Genetic Variants in *RKIP* Are Associated with Clear Cell Renal Cell Carcinoma Risk in a Chinese Population

**DOI:** 10.1371/journal.pone.0109285

**Published:** 2014-10-16

**Authors:** Qiang Cao, Jian Wang, Mingcong Zhang, Pu Li, Jian Qian, Shaobo Zhang, Lei Zhang, Xiaobing Ju, Meilin Wang, Zhengdong Zhang, Jie Li, Min Gu, Wei Zhang, Chao Qin, Pengfei Shao, Changjun Yin

**Affiliations:** 1 State Key Laboratory of Reproductive Medicine, Department of Urology, the First Affiliated Hospital of Nanjing Medical University, Nanjing, China; 2 Department of oncology, the First Affiliated Hospital of Nanjing Medical University, Nanjing, China; 3 Cancer Center of Nanjing Medical University, Department of Molecular & Genetic Toxicology, Nanjing Medical University, Nanjing, China; National Cancer Center, Japan

## Abstract

**Background:**

Raf-1 kinase inhibitor protein (RKIP) plays a critical role in tumor development by regulating cell functions such as invasion, apoptosis and differentiation. Down-regulation of RKIP expression has been implicated in the development and progression of renal cell carcinoma (RCC). Herein, we hypothesized that genetic polymorphisms in *RKIP* might be associated with susceptibility and progression of RCC.

**Methods:**

A total of 5 tagging single-nucleotide polymorphisms (tSNPs) in *RKIP* were selected and genotyped by SNapShot method in a case-control study of 859 RCC patients and 1004 controls. The logistic regression was used to evaluate the genetic association with occurrence and progression of RCC. The functionality of the important SNP was preliminary examined by qRT-PCR.

**Result:**

We found that the rs17512051 in the promoter region of *RKIP* was significantly associated with decreased clear cell RCC (ccRCC) risk (TA/AA vs. TT: *P* = 0.039, OR = 0.78, 95%CI = 0.62–0.99). Another SNP (rs1051470) in the 3′UTR region of *RKIP* was marginally associated with increased ccRCC risk (TT vs. CC+CT: OR = 1.45, 95%CI = 1.01–2.09). In the stratified analysis, the protective effect of rs17512051 was more predominant in the subgroups of male, non-smokers, non-drinkers as well as subjects without history of diabetes. Furthermore, we observed higher *RKIP* mRNA levels in the presence of the rs17512051A allele in normal renal tissues.

**Conclusion:**

Our results suggest that the potentially functional *RKIP* rs17512051 polymorphism may affect ccRCC susceptibility through altering the endogenous *RKIP* expression level. Risk effects and the functional impact of this polymorphism need further validation.

## Introduction

Kidney cancer is the 13^th^ most common cancer globally. Every year, about 270,000 cases are diagnosed as kidney cancer while 116,000 die from this disease [1]. Clear cell renal cell carcinoma (ccRCC) is a major pathologic form, which accounts for about 90% of kidney malignancies. RCC has a higher incidence in western communities, and in China, about 37.7 men and 16.6 women per 100 000 individuals are diagnosed with RCC every year [2]. In addition to geographic variation, several other epidemiological risk factors have been established in sporadic RCC, including obesity, hypertension, smoking, alcohol intake and so on [3]. However, some people will not develop RCC although they are exposed to these risk factors during their lifetime. Accumulation of studies indicated that both environmental and genetic factors played critical roles in the tumorigenesis of RCC [4].

As we know, dysregulation of cell growth is closely related to cancer development and progression in various types of malignancies. Raf-1 kinase inhibitor protein (RKIP), which is known as one of the phosphatidylethanolamine-binding proteins (PEBP) family [5], has been reported to regulate growth and differentiation in a variety of species [6]. Emerging studies have indicated that RKIP functions as an intracellular regulator via several important signal cascades, including Raf-MEK-MAPK, G protein coupled receptor (GPCR) [7] and NF-κB pathway [8]. Further studies have shown that through these pathways, RKIP works as a multifunctional protein in carcinogenesis such as invasion, dysregulation of apoptosis and cell differentiation [9]–[12]. In 2002, Keller *et al.* discovered that the expression of RKIP is lower in metastatic prostate cancer cells than that in non-metastatic cells [13]. Subsequently, more and more reports have demonstrated down-regulation of RKIP in various cancers including melanoma [14], breast cancer [15], hepatocellular carcinoma [16], [17] and colorectal cancer [18]. In further studies, decreased level of RKIP has been revealed to be related with poor prognosis in prostate cancer, bladder cancer, and colorectal cancer [19]–[21]. Collectively, these researches suggest that RKIP may act as not only an inhibitor of tumorigenesis, but also a metastasis suppressor. In addition, Moon and his colleagues suggested that RKIP expression was a significant prognostic marker for RCC and its level was tightly associated with progression and metastasis in renal cancer [22]. Recently, a study of 310 RCC cases has suggested RKIP was a significant prognostic marker because of its close correlation with progression and metastasis of RCC. In the same study, researchers also found that reduced RKIP expression was related to higher disease stage, larger tumor size, sarcomatoid subtype and poor overall survival [23].

Considering the critical roles of RKIP played in carcinogenesis, it is possible that single nucleotide polymorphisms (SNPs) located in the functional regions of *RKIP* may play a role in the development of RCC by influencing the expression RKIP. To test our hypothesis, we selected 5 tagging SNPs (tSNPs) in *RKIP* and genotyped these tSNPs using SNaPshot method in a case-control study in Chinese population.

## Methods

### Ethics statement

The study was approved by the Institutional Review Board of the Nanjing Medical University, Nanjing, China. At recruitment, written informed consents were obtained from all participants involved in this study.

### Study population

Overall, in this case-control study 859 clear cell renal cell carcinoma cases and 1004 cancer-free controls were enrolled. All subjects are ethnic Han Chinese from different families and have no blood relationship in our study. All these cases were histopathologically confirmed to be incident ccRCC first time without prior history of other cancers, chemotherapy or radiotherapy, and they were consecutively recruited without restriction of age and sex from May 2004 at The First Affiliated Hospital of Nanjing Medical University, Nanjing, China. We classified the disease according to World Health Organization (WHO) criteria and staged it according to the American Joint Committee on Cancer (AJCC) TNM (tumor–node–metastasis) classification. The Fuhrman scale was used to assess tumor nuclear grade. All 1004 controls were enrolled from healthy subjects seeking routine physical examination in the outpatient departments at the same hospital and were frequency matched to the cases on sex and age (±5 years). All the controls were genetically unrelated to the cases and had no individual history of any cancers. Before recruitment, we administered a standard questionnaire containing demographic data and exposure information for every cases and controls by face-to-face interviews. Each of them donated 5 ml venous blood into the EDTA tube after providing a written informed consent. The response rate for both case and control subjects was >85%.

### SNP selection

**Figure 1:**
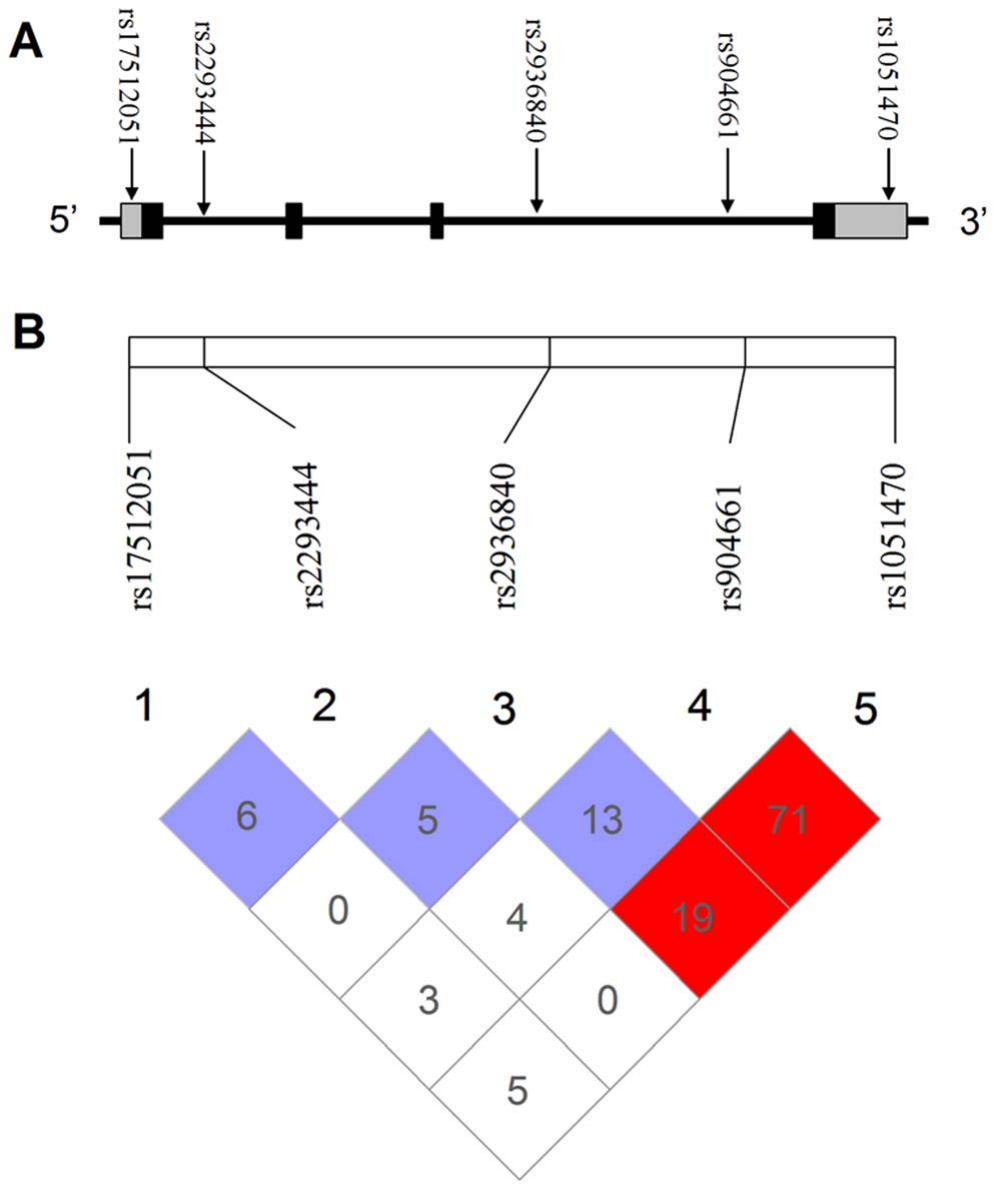
Genetic location and linkage disequilibrium (LD) of the five tag-SNPs. (A) The location of the five tag-SNPs in the RKIP gene. The exons were indicated by black boxes; the 5′ and 3′ untranslated regions were denoted by gray boxes. (B). LD plot among the five tag-SNPs in the RKIP gene (data from HapMap).

The SNPs were selected by using the genotype data obtained from unrelated Han Chinese in Beijing individuals in the HapMap database (HapMap Data Rel 21a/Phase II, Jan07, on NCBI B35 assembly, dbSNP b125). We reviewed all the SNPs that had a minor allele frequency >5% in Han Chinese in Beijing within a 9.9 kb region spanning the *RKIP* gene including 2 kb upstream and 2 kb downstream (shown in Table S1). The identification of the tag-SNPs was using the pairwise option of the Haploview 4.2 software and an r^2^ of 0.8 was selected as a threshold for the analyses. As a result, 5 tag-SNPs (rs17512051, rs1051470, rs2293444, rs904661 and rs2936840) were selected at a resolution of one SNP per 1.98 kb that captured all variant alleles with a mean r^2^ of 0.963. The identification number and relative position of the 5 tag-SNPs as well as the LD plot of the tag-SNPs presented by the Haploview 4.2 software are shown in Figure 1.

### DNA extraction and genotyping

The whole genomic DNA was extracted from the peripheral blood leukocyte by proteinase K digestion and phenol-chloroform extraction (QIAamp DNA Blood Kit). In this study, we genotyped 5 tSNPs using SNapShot assay according to the manufacturer's instructions (Applied Biosystems). The primers to amplify different fragments containing each SNP were designed using Primer3 software. Briefly, the reaction was performed in 40 uL volumn containing 2 uL extracted genomic DNA, 1 uL of 50 mM MgCl2, 1 uL of 10 mM dNTP, 4 uL of 10×Buffer, 1 uL of primer mix and 0.4 uL of 5U Platinum Taq DNA Polymerase in 30 uL of Dnase-free water. The amplification program was performed in the start of 95°C for 2 min, followed by 33 cycles of 94°C for 20 sec, 56°C for 30 sec and then 72°C for 40 sec. We performed the Shrimp Alkaline Phosphatase Method to purify the PCR product. In the procedure, 0.3 uL of SAP and 0.2 uL of Exonuclease I were mixed with 2 uL PCR product in 7.5 uL of ddH_2_O at 37°C for 100 min followed by 15 min in 75°C. The extention reaction was then carried out in 5 uL volumn containing 2.5 uL of Reaction Mix (Applied Biosystems), 1 uL of Probe Mix and 1.5 uL of PCR pruduct and performed in 25 cycles of 96°C, 51°C, 60°C sequentially for 10 s, 5 s and 30 s. And finally, the extention products were purified by Shrimp Alkaline Phosphatase Method in 0.5 uL of SAP and 2.5 uL of ddH_2_O in 37°C for 1 h and 75°C for 15 min to inactive SAP. The final purified extention products were analyzed in 96-well PRISM 3730 DNA Sequencer (Applied Biosystems). All our procedure of genotyping was carried out in double-blind control. And in addition, 5% of the samples were selected randomly for confirmation and the results were 100% concordant.

### Analysis of *RKIP* expression

A total of 42 paratumor renal tissues were used to analyze *RKIP* mRNA levels in vivo. The tissues were taken from the surgically removed samples from the patients and were immediately stored in liquid nitrogen. Total RNA was isolated from about 100 mg frozen tissue with TRIzol (Invitrogen, Carlsbad, CA, USA) and reverse transcribed to single stranded cDNA using an oligo (dT) primer and Superscript II (Invitrogen) following the manufacturer's protocol. The *RKIP* mRNA level was measured by quantitative real-time reverse transcription (RT)-PCR on the ABI Prism 7900 sequence detection system (Applied Biosystems, Foster City, CA, USA). The glyceraldehyde-3-phosphate dehydrogenase (GAPDH) was used as an internal reference gene. The primers used for *RKIP* were 5′- CAATGACATCAGCAGTGGCACAGTC (forward) and 5′-CACAAGTCATCCCACTCGGCCTG-3′ (reverse); and for GAPDH were 5′-GCACCGTCAAGGCTGAGAAC-3′ (forward) and 5′- TGGTGAAGACGCCAGTGGA-3′ (reverse). The reaction mixture contained 0.1 M of each primer, 26SYBR Green PCR Master Mix (TaKaRa, Berkeley, CA, USA), and 1 mL of cDNA (1∶10 dilution). The amplification was performed under the following conditions: 95°C for 30 s, and 40 cycles of 95°C for 15 s and 60°C for 30 s. Each reaction was done in triplicate.

### Statistical analysis

We used the Pearson's chi-square test (for categorical variables) and the student's t-test (for continuous variables) to compare the differences in variables such as gender, smoking status, drinking status, and the frequency distribution of every genotypes between the cases and controls. A goodness-of-fit chi-square test was used to assess Hardy-Weinberg equilibrium (HWE) for each 5 tSNPs. Computing odds ratios (ORs) and 95% confidence intervals (CIs) were conducted to estimate the associations between polymorphisms and risk of ccRCC with adjusted data. Differences in *RKIP* mRNA levels from renal tissues carrying different genotypes were evaluated by Student's t test or one-way ANOVA test. All data were analyzed with the software SAS 9.1.3 (SAS Institute, Cary, NC, USA) with two-sided test, and the adjusted P value of less than 0.05 was considered to be statistically significant.

## Result

### Characteristics of the Study Cases and Controls

The frequency distributions of the selected characteristics of 859 cases and 1004 controls are shown in Table 1. No significant differences regarding to age (*P* = 0.774), gender (*P* = 0.102) and drinking status (*P* = 0.113) were observed among the patients and controls. However, there were more hypertension patients, smokers and diabetics among the cases than among the controls (all *P*≤0.001), and the BMI level in cases was statistically higher than that in controls (*P* = 0.007). In our study, 67.2% of the patients were clinical stage I, and 19.8%, 6.6% and 6.4% were found to be stage II, III, IV, respectively. The percentage of nuclear grades from I to IV was 20.5%, 54.7%, 19.9%, and 4.9%, respectively.

**Table 1:** Frequency distribution of selected variables between the clear cell renal cell carcinoma cases and control subjects.

Variables	Cases (n = 859)		Controls (n = 1004)		*P*-value^a^
	N	%	N	%	
Age (years) (mean ± SD)	57.0±11.7		57.2±12.4		0.774
BMI (kg/m2) (mean ± SD)	24.2±2.9		23.8±3.1		**0.007**
Gender					
Male	557	64.8	687	68.4	0.102
Female	302	35.2	317	31.6	
Smoking status					
Never	530	61.7	665	66.2	**0.001**
Former	140	16.3	104	10.4	
Current	189	22.0	235	23.4	
Drinking status					
Never	619	72.1	756	75.3	0.113
Ever	240	27.9	248	24.7	
Hypertension					
No	522	60.8	675	67.2	**<0.001**
Yes	337	39.2	251	25.0	
Withdraw	0	0	78	7.8	
Diabetes					
No	748	87.1	870	86.7	**<0.001**
Yes	111	12.9	56	5.6	
Withdraw	0	0	78	7.8	
Clinical stage					
I	577	67.2			
II	170	19.8			
III	57	6.7			
IV	55	6.4			
Grade					
I	176	20.5			
II	470	54.7			
III	171	19.9			
IV	42	4.9			

a Student's t-test for age and BMI distributions between cases and controls; two-sided χ2-test for others selected variables between cases and controls.

### The Association between the *RKIP* Polymorphisms and Risk of Clear Cell Renal Cell Carcinoma

The characteristics of the selected tSNPs are presented in Table 2. The allele frequencies of rs904661 in control group did not conform to HWE, and therefore was excluded from further analysis. The detailed genotype and allele distributions of the remaining 4 tSNPs in the patients and controls are presented in Table 3. No significant difference in genotype and allele distributions of rs2293444 and rs2936840 were observed between the cases and controls (*P* = 0.132 and 0.836, respectively). We found that the rs17512051 in the 5′UTR region of *RKIP* was significantly associated with ccRCC risk. Compared with individuals with homozygote TT genotype, those subjects carrying the variant genotypes (TA/AA) had a significant decreased ccRCC risk (OR = 0.78, 95%CI = 0.62–0.99). Significant association was also observed in allele comparison (OR = 0.79, 95%CI = 0.65–0.97). Another SNP (rs1051470), located in the 3′UTR region of *RKIP* was also found to be associated with ccRCC risk, although the association was less robust. For this SNP, significant association was only observed in the dominant model (TT vs. CC+CT, OR = 1.45, 95%CI = 1.01–2.09). No significant difference in allele frequency distribution was observed between cases and controls (*P* = 0.306).

**Table 2:** The characteristics of the 5 tSNPs in *RKIP* gene.

Polymorphism	Alleles	Location	MAF	HWE^a^
rs17512051	T>A	promoter	0.119	0.611
rs1051470	C>T	3′UTR	0.331	0.102
rs2293444	A>C	intron	0.298	0.115
rs904661	C>T	intron	0.391	**0.005**
rs2936840	C>T	intron	0.190	0.062

a Goodness-of-fit chi-square test was used to assess Hardy–Weinberg equilibrium (HWE) in controls.

**Table 3:** Association between the 4 tSNPs in *RKIP* and risk of clear cell renal cell carcinoma.

Polymorphisms	Cases, n (%)	Controls, n (%)	*P* a	Adjusted OR (95% CI)^b^
Rs17512051				
TT	669 (80.2)	752 (76.2)	0.081	1.00 (reference)
TA	156 (18.7)	217 (22.0)		0.80 (0.63–1.02)
AA	9 (1.1)	18 (1.8)		0.52 (0.23–1.18)
TA/AA	165 (19.8)	235 (23.8)	**0.039**	**0.78 (0.62–0.99)**
T allele	1494	1721		1.00 (reference)
A allele	174	253	**0.026**	**0.79 (0.65–0.97)**
Rs1051470				
CC	410 (51.3)	501 (51.6)	0.084	1.00 (reference)
CT	316 (39.5)	407 (41.9)		0.98 (0.80–1.20)
TT	74 (9.2)	63 (6.5)		1.42 (0.98–2.06)
CT+TT	390 (48.75)	470 (48.41)	0.885	1.04 (0.86–1.27)
CC+CT	726 (90.8)	908 (93.5)		1.00 (reference)
TT	74 (9.2)	63 (6.5)	**0.044**	**1.45 (1.01–2.09)**
C allele	1136	1409		1.00 (reference)
T allele	464	533	0.306	1.08 (0.93–1.25)
Rs2293444				
AA	219 (26.9)	262 (27.3)	0.132	1.00 (reference)
AC	398 (48.8)	502 (52.3)		0.98 (0.78–1.24)
CC	198 (24.3)	196 (20.4)		1.28 (0.97–1.69)
AC+CC	596 (73.1)	698 (72.7)	0.843	1.06 (0.85–1.32)
A allele	836	1026	0.201	1.00 (reference)
C allele	794	894		1.09 (0.96–1.24)
Rs2936840				
CC	699 (84.4)	832 (84.7)	0.836	1.00 (reference)
CT	117 (14.1)	139 (14.2)		0.96 (0.73–1.27)
TT	12 (1.5)	11 (1.1)		1.12 (0.48–2.60)
CT+TT	129 (15.6)	150 (15.3)	0.858	0.97 (0.75–1.26)
C allele	1515	1803	0.731	1.00 (reference)
T allele	141	161		1.04 (0.82–1.32)

a Two-sided χ2-test for the distributions of either genotype or allele frequencies between the cases and controls.

b genotype-specific ORs were adjusted for age, gender, BMI, smoking status, drinking status, diabetes and hypertension in logistic regression model.

### Stratification analyses of *RKIP* rs17512051 and risk of RCC

We then further evaluated the effect of *RKIP* rs17512051 on RCC occurrence stratified by age, gender, BMI, smoking status, drinking status, hypertension and diabetes. As shown in Table 4, the association between *RKIP* rs17512051 and RCC risk appeared stronger in subgroups of males (*P* = 0.016, OR = 0.70, 95%CI = 1.15–3.21), non-smokers (*P* = 0.033, OR = 0.73, 95%CI = 0.54–0.97), non-drinkers (*P* = 0.041, OR = 0.75, 95%CI = 0.57–0.99) and individuals without history of diabetes (*P* = 0.021, OR = 0.75, 95%CI = 0.59–0.96). Since the significance of *RKIP* rs1051470 was less robust, and the number of individuals with rs1051470TT was small (74 and 63 in cases and controls, respectively), which would be further reduced in the stratified analysis, and could cause unstable associations. Therefore, we will not discuss the stratified analysis of *RKIP* rs1051470 in the study, but the results are presented in Table S2.

**Table 4:** Stratification analyses between the *RKIP* rs17512051 polymorphisms and risk of clear cell renal cell carcinoma.

Variables	*RKIP* rs17512051 genotypes				*P* a	Adjusted OR (95% CI)^a^
	TT (n, %)		TA/AA (n, %)			
	Case (n, %)	Control (n,%)	Case (n, %)	Control (n, %)		
Age						
≤57	347 (80.5)	395 (74.7)	84 (19.5)	134 (25.3)	0.057	0.73 (0.54–1.01)
>57	322(79.9)	357 (78.0)	81 (20.1)	101 (22.0)	0.413	0.86 (0.61–1.22)
BMI						
≤24	321 (79.7)	410 (75.8)	82 (20.4)	131 (24.2)	0.242	0.82 (0.59–1.14)
>24	348 (80.7)	342 (76.7)	83 (19.3)	104 (23.3)	0.080	0.74 (0.53–1.03)
Gender						
Male	434 (80.4)	506 (75.0)	106 (19.6)	169 (25.4)	**0.016**	**0.70 (0.53–0.94)**
Female	235 (79.9)	246 (78.9)	59 (20.1)	66 (21.2)	0.843	0.96 (0.64–1.43)
Smoking						
Never	424 (82.1)	502 (76.6)	93 (18.0)	153 (23.4)	**0.033**	**0.73 (0.54–0.97)**
Ever	245 (77.3)	250 (75.3)	72 (22.7)	82 (24.7)	0.821	0.96 (0.65–1.41)
Drinking						
Never	489 (81.0)	570 (76.6)	115 (19.0)	174 (23.4))	**0.041**	**0.75 (0.57–0.99)**
Ever	180 (78.3)	182 (74.9)	50 (21.7)	61 (25.1)	0.376	0.81 (0.52–1.28)
HBP						
No	413 (81.0)	564 (76.3)	97 (19.0)	175 (23.7)	0.064	0.76 (0.57–1.01)
Yes	256 (79.0)	248 (76.5)	68 (21.0)	76 (23.4)	0.450	0.81 (0.54–1.21)
Diabetes						
No	581 (80.4)	706 (75.8)	142 (19.6)	226 (24.2)	**0.021**	**0.75 (0.59–0.96)**
Yes	88 (79.3)	106 (80.9)	23 (20.7)	25 (19.1)	0.675	1.21 (0.50–2.95)

a Adjusted for age, gender, BMI, smoking status, drinking status, hypertension and diabetes in logistic regression model.

### The influence of polymorphisms on *RKIP* expression

**Figure 2:**
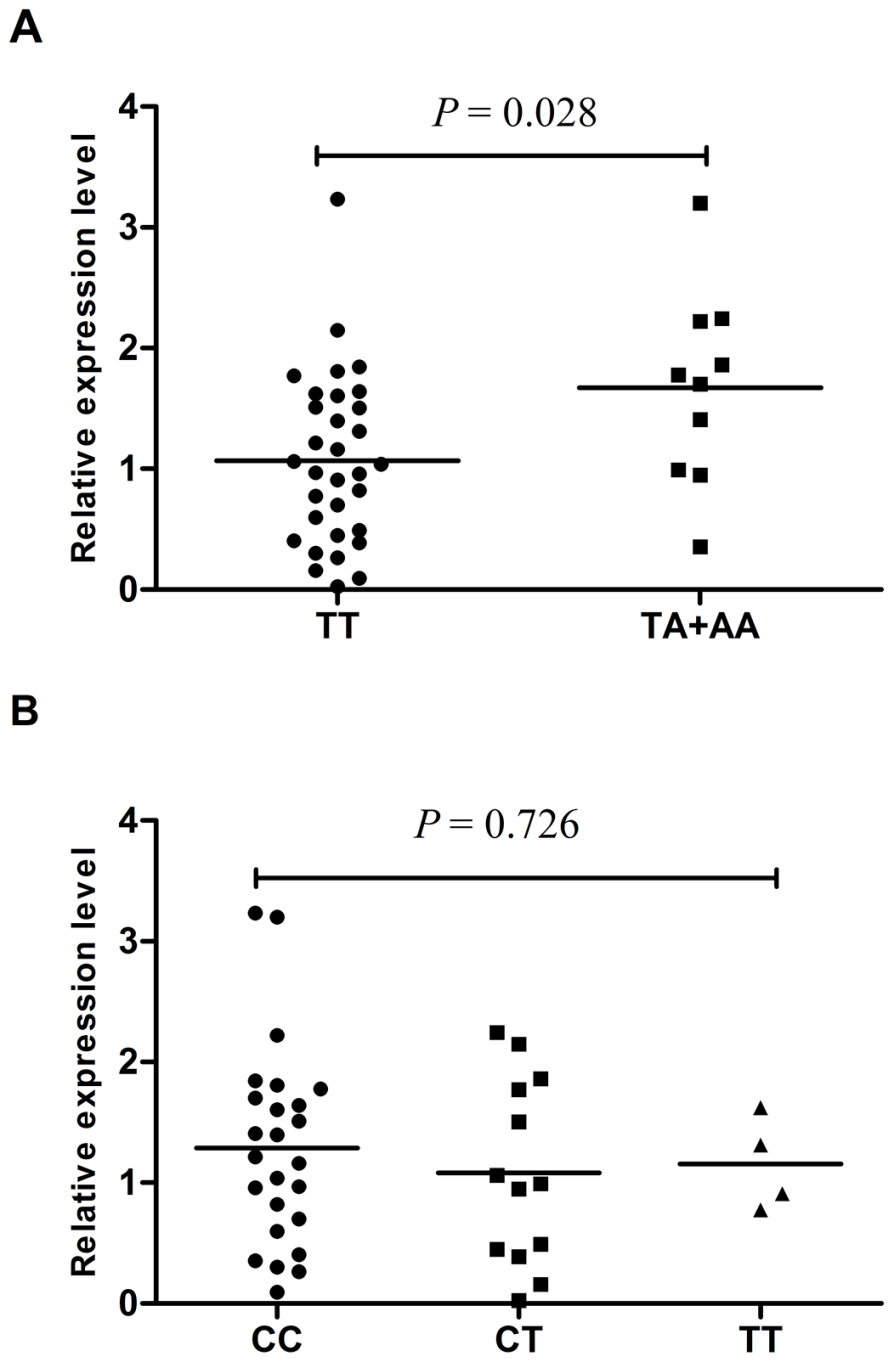
Expression of *RKIP* in cancer adjacent normal renal tissues. (A), Association between the *RKIP* expression in renal tissues and *RKIP* rs17512051 genotypes. The *RKIP* rs17512051 TA/AA genotype is associated with significantly higher *RKIP* expression than the *RKIP* rs17512051TT genotype (*P* = 0.028). (B), Association between the *RKIP* expression in renal tissues and *RKIP* rs1051470 genotypes. No significant differences in *RKIP* expression among different *RKIP* rs1051470 genotypes were observed (*P* = 0.726).

Since the two *RKIP* rs17512051 and rs1051470 are located in the functional region of *RKIP*, we then examined whether they had impact on *RKIP* expression using real-time quantitative RT-PCR. As shown in Figure 2, the preliminary data showed that the renal tissues with *RKIP* rs17512051 TA/AA genotypes had higher level of RKIP expression, compared with tissues carrying the TT genotype (*P* = 0.028). However, no significant difference in RKIP expression was observed among renal tissue with different *RKIP* rs1051470 genotypes (*P* = 0.726).

### Association between the *RKIP* rs17512051 polymorphism and the clinicopathological characteristics of ccRCC patients

As expression of RKIP was reported to be involved in the progression of RCC, we then investigated whether the *RKIP* rs17512051 polymorphism has association with the clinicopathological parameters of ccRCC patients. However, as presented in Table 5, the difference in the distribution of rs17512051 genotype among different stage and grade of ccRCC was not significant (*P* = 0.134 and 0.440, respectively).

**Table 5:** The association of *RKIP* rs17512051 polymorphism and clinical stage and tumor grade of ccRCC patients.

Category	TT (n, %)	TA/AA (n, %)	*P* *	Adjusted OR (95% CI)†
Clinical Stage				
I	460 (68.8)	101 (61.2)	0.134	1.00 (reference)
II	124 (18.5)	40 (24.2)		1.44 (0.94–2.20)
III	45 (6.7)	9 (5.5)		0.91 (0.43–1.95)
IV	40 (6.0)	15 (9.1)		1.54 (0.81–2.93)
Tumor grade				
I	144 (21.5)	28 (17.0)	0.440	1.00 (reference)
II	357 (53.4)	96 (58.2)		1.37 (0.86–2.19)
III	133 (19.9)	35 (21.2)		1.32 (0.76–2.31)
IV	35 (5.2)	6 (3.6)		0.77 (0.29–2.05)

*Two-sided χ2-test.

† ORs were adjusted for age, sex, BMI, smoking, drinking status, diabetes and hypertensionin logistic regression model. CI: confidence interval; OR: odds ratio.

## Discussion

In the present study, we investigated the associations between 4 tSNPs in *RKIP* and RCC susceptibility in a Chinese population. Our study suggested that the *RKIP* rs17512051 was associated with a decreased risk of ccRCC. The association study results of rs17512051 were subsequently confirmed by a preliminary functional analysis of the variant, in which we observed that the *RKIP* mRNA level was increased in individuals who carried the rs17512051A allele. Besides, we also found rs105147 in the 3′UTR region of *RKIP* was associated with increased RCC risk; however, the significant was less robust, and it seems this polymorphism does not have impact on the expression of *RKIP*.

As a tumor suppressor, Raf-1 kinase inhibitor protein (RKIP) functions as a multi-aspect factor to inhibit various cellular processes including cell differentiation, cell cycle, cell migration and apoptosis [24]. Collective evidence from researches indicates that the decreased expression level of RKIP can trigger initiation of cancer and even metastasis [14]–[18]. And in RCC, down-regulation of RKIP was reported to be involved in the renal carcinogenesis and was also a significant prognostic marker for RCC patients [22]. However, there is lack of study on the association between genetic variants in *RKIP* and susceptibility of RCC, and to our best knowledge, this is the first report to evaluate the above-mentioned associations.

Considering the polymorphism of rs17512051 is located in the promoter of *RKIP*, it is predicted that the *RKIP* rs17512051 variant may lead to abnormal expression of *RKIP*. In addition, according to the UCSC website, dbSNP website in NCBI and the software of TFSEARCH, we deduce that the change of rs17512051T allele to rs17512051A may create a new transcription factor binding site of Cap. Alternatively, when the allele site of SNP rs17512051 is “A”, a new transcription factor of Cap can bind to the promoter region to increase the transcription and expression of *RKIP* which may induce a higher RKIP expression in the subjects carrying the rs17512051A allele. Therefore, it is biologically plausible that *RKIP* rs17512051 has an effect on the risk of ccRCC.

Furthermore, through stratification analyses, we observed that the association between *RKIP* rs17512051 and reduced ccRCC risk was more prominent in males, suggesting that the interaction of gender and genetic variants may contribute to the occurrence of ccRCC together. We also found that the decreased risk was more pronounced in subjects without history of diabetes although this is established factors for ccRCC occurrence, indicating that the effect of the polymorphism might be overwhelmed by effect of plasma glucose on ccRCC. Interestingly, the association was stronger in non-smokers and non-drinkers, suggesting that environmental factors might be more predominant than genetic cause of this polymorphism. Aberrant expression of *RKIP* was reported to contribute to the progression of RCC; however we did not find any significant association between *RKIP* rs17512051 and ccRCC stage and grade. We speculate that the influence of rs17512051 on *RKIP* expression is relatively subtle that may slowly affect the development of ccRCC, but not power enough to influence the disease progression.

Since our case-control study was hospital based, we couldn't rule out a possibility of selection bias of these subjects who might be associated with a particular genotype. In our study, except the rs904661, which had been excluded, the other 4 tSNPs all conformed to HWE. The genotype distributions of the 4 tSNPs in the controls were similar to that in the HapMap database of Han Chinese population. Therefore, the selection bias in terms of genotype distributions could be unsubstantial.

## Conclusion

In summary, our result suggests that the potentially functional *RKIP* rs17512051 polymorphism may affect ccRCC susceptibility through altering the endogenous *RKIP* expression level. More detailed investigations and further studies on genetic functions are required in the future.

## Supporting Information

Table S1 **All the single nucleotide polymorphism that were reviewed in the presented study.**
(DOC)

Table S2 **Stratification analyses between the** ***RKIP*** **rs1051470 polymorphism and risk of clear cell renal cell carcinoma.**
(DOC)

## References

[1] Ljungberg B, Campbell SC, Choi HY, Jacqmin D, Lee JE, et al (2011) The epidemiology of renal cell carcinoma. Eur Urol 60: 615–621. 21741761 10.1016/j.eururo.2011.06.049

[2] Yang L, Parkin DM, Ferlay J, Li L, Chen Y (2005) Estimates of cancer incidence in China for 2000 and projections for 2005. Cancer Epidemiol Biomarkers Prev 14: 243–250. 15668501

[3] Chow WH, Dong LM, Devesa SS (2010) Devesa, Epidemiology and risk factors for kidney cancer. Nat Rev Urol 7: 245–257. 20448658 10.1038/nrurol.2010.46 PMC3012455

[4] Semenza JC, Ziogas A, Largent J, Peel D, Anton-Culver H (2001) Gene-environment interactions in renal cell carcinoma. Am J Epidemiol 153: 851–859. 11323315 10.1093/aje/153.9.851

[5] Seddiqi N, Bollengier F, Alliel PM, Périn JP, Bonnet F, et al (1994) Amino acid sequence of the Homo sapiens brain 21–23-kDa protein (neuropolypeptide h3), comparison with its counterparts from Rattus norvegicus and Bos taurus species, and expression of its mRNA in different tissues. J Mol Evol 39: 655–650. 7807553 10.1007/BF00160411

[6] Trakul N, Rosner MR (2005) Rosner, Modulation of the MAP kinase signaling cascade by Raf kinase inhibitory protein. Cell Res 15: 19–23. 15686621 10.1038/sj.cr.7290258

[7] Lorenz K, Lohse MJ, Quitterer U (2003) Quitterer, Protein kinase C switches the Raf kinase inhibitor from Raf-1 to GRK-2. Nature 426: 574–579. 14654844 10.1038/nature02158

[8] Yeung KC, Rose DW, Dhillon AS, Yaros D, Gustafsson M, et al (2001) Raf kinase inhibitor protein interacts with NF-kappaB-inducing kinase and TAK1 and inhibits NF-kappaB activation. Mol Cell Biol 21: 7207–7217. 11585904 10.1128/MCB.21.21.7207-7217.2001 PMC99896

[9] Akaishi J, Onda M, Asaka S, Okamoto J, Miyamoto S, et al (2006) Growth-suppressive function of phosphatidylethanolamine-binding protein in anaplastic thyroid cancer. Anticancer Res 26: 4437–4442. 17201166

[10] Zhang L, Fu Z, Binkley C, Giordano T, Burant CF, et al (2004) Raf kinase inhibitory protein inhibits beta-cell proliferation. Surgery 136: 708–715. 15349122 10.1016/j.surg.2003.12.013

[11] Hellmann J, Rommelspacher H, Mühlbauer E, Wernicke C (2010) Raf kinase inhibitor protein enhances neuronal differentiation in human SH-SY5Y cells. Dev Neurosci 32: 33–46. 19955695 10.1159/000236595

[12] Zhu S, Mc Henry KT, Lane WS, Fenteany G (2005) A chemical inhibitor reveals the role of Raf kinase inhibitor protein in cell migration. Chem Biol 12: 981–991. 16183022 10.1016/j.chembiol.2005.07.007

[13] Fu Z, Dozmorov IM, Keller ET (2002) Osteoblasts produce soluble factors that induce a gene expression pattern in non-metastatic prostate cancer cells, similar to that found in bone metastatic prostate cancer cells. Prostate 51: 10–20. 11920953 10.1002/pros.10056

[14] Schuierer MM, Bataille F, Hagan S, Kolch W, Bosserhoff AK (2004) Reduction in Raf kinase inhibitor protein expression is associated with increased Ras-extracellular signal-regulated kinase signaling in melanoma cell lines. Cancer Res 64: 5186–5192. 15289323 10.1158/0008-5472.CAN-03-3861

[15] Hagan S, Al-Mulla F, Mallon E, Oien K, Ferrier R, et al (2005) Reduction of Raf-1 kinase inhibitor protein expression correlates with breast cancer metastasis. Clin Cancer Res 11: 7392–7397. 16243812 10.1158/1078-0432.CCR-05-0283

[16] Schuierer MM, Bataille F, Weiss TS, Hellerbrand C, Bosserhoff AK (2006) Raf kinase inhibitor protein is downregulated in hepatocellular carcinoma. Oncol Rep 16: 451–456. 16865242

[17] Lee HC, Tian B, Sedivy JM, Wands JR, Kim M (2006) Loss of Raf kinase inhibitor protein promotes cell proliferation and migration of human hepatoma cells. Gastroenterology 131: 1208–1217. 17030190 10.1053/j.gastro.2006.07.012 PMC2593881

[18] Minoo P, Zlobec I, Baker K, Tornillo L, Terracciano L, et al (2007) Loss of raf-1 kinase inhibitor protein expression is associated with tumor progression and metastasis in colorectal cancer. Am J Clin Pathol 127: 820–827. 17439843 10.1309/5D7MM22DAVGDT1R8

[19] Fu Z, Kitagawa Y, Shen R, Shah R, Mehra R, et al (2006) Metastasis suppressor gene Raf kinase inhibitor protein (RKIP) is a novel prognostic marker in prostate cancer. Prostate 66: 248–256. 16175585 10.1002/pros.20319

[20] Al-Mulla F, Hagan S, Behbehani AI, Bitar MS, George SS, et al (2006) Raf kinase inhibitor protein expression in a survival analysis of colorectal cancer patients. J Clin Oncol 24: 5672–5679. 17179102 10.1200/JCO.2006.07.5499

[21] Afonso J, Longatto-Filho A, Martinho O, Lobo F, Amaro T, et al (2013) Low RKIP expression associates with poor prognosis in bladder cancer patients. Virchows Arch 462: 445–453. 23462986 10.1007/s00428-013-1388-2

[22] Moon A, Park JY, Sung JY, Park YK, Kim YW (2012) Reduced expression of Raf-1 kinase inhibitory protein in renal cell carcinoma: a significant prognostic marker. Pathology 44: 534–539. 22935977 10.1097/PAT.0b013e32835817e8

[23] Moon A, Park JY, Sung JY, Park YK, Kim YW (2012) Reduced expression of Raf-1 kinase inhibitory protein in renal cell carcinoma: a significant prognostic marker. Pathology 44: 534–539. 22935977 10.1097/PAT.0b013e32835817e8

[24] Zeng L, Imamoto A, Rosner MR (2008) Raf kinase inhibitory protein (RKIP): a physiological regulator and future therapeutic target. Expert Opin Ther Targets 12: 1275–1287. 18781826 10.1517/14728222.12.10.1275

