# Supplementary material for: Genetic Variants in RKIP Are Associated with Clear Cell Renal Cell Carcinoma Risk in a Chinese Population
Source: PLoS One. 2014 Oct 16;9(10):e109285. doi: 10.1371/journal.pone.0109285 (PMC4199597; doi:10.1371/journal.pone.0109285)
Supplement: Table S1 — All the single nucleotide polymorphism that were reviewed in the presented study. (DOC) [file pone.0109285.s001.doc]

| Number | Name | Position | MAF | Alleles |
| --- | --- | --- | --- | --- |
| 1 | rs12312445 | 117057281 | 0 | A:A |
| 2 | rs17512051 | 117058050 | 0.067 | T:A |
| 3 | rs2293444 | 117058565 | 0.478 | A:C |
| 4 | rs11068843 | 117058591 | 0 | C:C |
| 5 | rs2088702 | 117059479 | 0.467 | C:T |
| 6 | rs7977921 | 117060993 | 0.451 | T:C |
| 7 | rs3741446 | 117061865 | 0 | C:C |
| 8 | rs2936840 | 117063226 | 0.056 | C:T |
| 9 | rs904662 | 117066216 | 0.205 | G:A |
| 10 | rs904661 | 117066243 | 0.3 | C:T |
| 11 | rs1050625 | 117066860 | 0 | C:C |
| 12 | rs11554096 | 117066975 | 0 | A:A |
| 13 | rs1051470 | 117067615 | 0.233 | C:T |
| 14 | rs1726407 | 117068109 | 0.244 | T:C |
| 15 | rs11068849 | 117069640 | 0.014 | C:T |
| 16 | rs7974375 | 117070081 | 0.023 | G:T |

**Table S1- All the single nucleotide polymorphism that were reviewed in the presented study**

MAF: minor allele frequency
