# Supplementary material for: Genetic Variants in RKIP Are Associated with Clear Cell Renal Cell Carcinoma Risk in a Chinese Population
Source: PLoS One. 2014 Oct 16;9(10):e109285. doi: 10.1371/journal.pone.0109285 (PMC4199597; doi:10.1371/journal.pone.0109285)
Supplement: Table S2 — Stratification analyses between the RKIP rs1051470 polymorphism and risk of clear cell renal cell carcinoma. (DOC) [file pone.0109285.s002.doc]

**Table S2-Stratification analyses between the *RKIP* rs1051470 polymorphism and risk of clear cell renal cell carcinoma**

| Variables | *RKIP* rs1051470 genotypes | | | | | *P* | Adjusted OR (95% CI) ^a^ |
| --- | --- | --- | --- | --- | --- | --- | --- |
|  | CC/CT (n, %) | |  | TT (n, %) | |  |  |
|  | Case (n, %) | Control (n,%) |  | Case (n, %) | Control (n, %) |  |  |
| Age |  |  |  |  |  |  |  |
| ≤57 | 397 (90.7) | 497 (94.0) |  | 40 (9.3) | 32 (6.0) | 0.059 | 1.67 (1.02-2.72) |
| >57 | 369 (91.6) | 428 (93.5) |  | 34 (8.4) | 30 (6.6) | 0.292 | 1.21 (0.70-2.08) |
| BMI |  |  |  |  |  |  |  |
| ≤24 | 369 (91.6) | 501 (92.6) |  | 34 (8.4) | 40 (7.4) | 0.555 | 1.03 (0.64-1.70) |
| >24 | 391 (90.7) | 424 (95.1) |  | 40 (9.3) | 22 (4.9) | 0.012 | 2.19 (1.23-3.89) |
| Gender |  |  |  |  |  |  |  |
| Male | 489 (90.6) | 635 (94.1) |  | 51 (9.4) | 40 (5.9) | 0.021 | 1.67 (1.06-2.62) |
| Female | 272 (92.2) | 290 (93.0) |  | 23 (7.8) | 22 (7.1) | 0.717 | 1.07 (0.57-2.00) |
| Smoking |  |  |  |  |  |  |  |
| Never | 469 (90.7) | 6145 (93.9) |  | 48 (9.3) | 40 (6.1) | 0.040 | 1.48 (0.95-2.31) |
| Ever | 291 (91.8) | 310 (93.4) |  | 26 (8.2) | 22 (6.6) | 0.443 | 1.49 (0.78-2.85) |
| Drinking |  |  |  |  |  |  |  |
| Never | 544 (90.1) | 693 (93.2) |  | 60 (9.9) | 51 (6.9) | 0.042 | 1.46 (0.98-2.19) |
| Ever | 216 (93.9) | 232 (95.5) |  | 14 (6.1) | 11 (4.5) | 0.448 | 1.28 (0.54-3.05) |
| HBP |  |  |  |  |  |  |  |
| No | 466 (91.4) | 694 (93.9) |  | 44 (8.6) | 45 (6.1) | 0.087 | 1.49 (0.96-2.34) |
| Yes | 294 (90.7) | 303 (93.5) |  | 30 (9.3) | 21 (6.5) | 0.188 | 1.35 (0.72-2.53) |
| Diabetes |  |  |  |  |  |  |  |
| No | 660 (91.3) | 874 (93.8) |  | 63 (8.7) | 58 (6.2) | 0.054 | 1.44 (0.98-2.11) |
| Yes | 100 (90.1) | 123 (93.9) |  | 11 (9.9) | 8 (6.1) | 0.273 | 1.45 (0.42-5.02) |

^a^ Adjusted for age, gender, BMI, smoking status, drinking status, hypertension and diabetes in logistic regression model.
